# Progress in the Study of Colorectal Cancer Caused by Altered Gut Microbiota After Cholecystectomy

**DOI:** 10.3389/fendo.2022.815999

**Published:** 2022-02-24

**Authors:** Yanpeng Ma, Ruize Qu, Yi Zhang, Changtao Jiang, Zhipeng Zhang, Wei Fu

**Affiliations:** ^1^ Department of General Surgery, Peking University Third Hospital, Beijing, China; ^2^ Cancer Center, Peking University Third Hospital, Beijing, China; ^3^ Department of Physiology and Pathophysiology, School of Basic Medical Sciences, Peking University, Beijing, China; ^4^ Key Laboratory of Molecular Cardiovascular Science (Peking University), Ministry of Education, Beijing, China; ^5^ Center of Basic Medical Research, Institute of Medical Innovation and Research, Third Hospital, Peking University, Beijing, China; ^6^ Center for Obesity and Metabolic Disease Research, School of Basic Medical Sciences, Peking University, Beijing, China

**Keywords:** colorectal cancer, gut microbiota, bile acid, genotoxin, diet, epidemiology, cholecystectomy

## Abstract

Epidemiological studies have found an increased incidence of colorectal cancer (CRC) in people who undergo cholecystectomy compared to healthy individuals. After cholecystectomy, bile enters the duodenum directly, unregulated by the timing of meals. Disruption of the balance of bile acid metabolism and increased production of primary bile acids, which in turn affects the composition and abundance of intestinal microorganisms. The link among cholecystectomy, the gut microbiota, and the occurrence and development of CRC is becoming clearer. However, due to the complexity of the microbial community, the mechanistic connections are less well understood. In this review, we summarize the changes of gut microbiota after cholecystectomy and illuminate the potential mechanisms on CRC, such as inflammation and immune regulation, production of genotoxins, metabolism of dietary ingredients, activation of signaling pathways, and so on. By reviewing these, we aimed to unravel the interactions between the gut microbiota and its host and be better positioned to develop treatments for CRC after cholecystectomy.

## Introduction

As one of the most common malignant tumors worldwide, colorectal cancer (CRC) ranks second and third in morbidity and mortality rates, respectively, and its incidence is gradually increasing in developing countries (1). It has been estimated that by 2030, the global disease burden of CRC will increase by 60%, and there will be more than 2.2 million new cases and 1.1 million deaths worldwide (2). Moreover, the morbidity and mortality rates of CRC are increasing yearly, particularly, in individuals under the age of 50 (3). Furthermore, there is continuous growth in the overall number of diagnosed cases, which contributes to the increasing disease burden of CRC (2). Therefore, research on the risk factors and pathogenesis of CRC is becoming increasingly essential.

CRC is associated with multiple factors, including genetic susceptibility and environmental factors, which play a greater role in its occurrence and development (4). The gut microbiota is among the various environmental factors recognized in cancer biology. There are more than 3×10^13^ bacterial cells in the human colorectum, which interact with host cells to regulate many physiological processes. Disruption of the gut microbiota affects the balance of physiological processes, contributing to the development and progression of many diseases, such as inflammatory bowel disease (IBD) and CRC (5–8).

Changes in lifestyle and eating habits are directly linked to an increase in gallbladder diseases (9). Although cholecystectomy is an acceptable standard treatment for gallbladder diseases (2016), there is evidence that it is likely to increase the incidence of CRC (10–13). However, little is known about the mechanisms responsible for this process. There are two major theories on the effects of cholecystectomy on CRC development (1): cholecystectomy may alter the concentration, composition, and excretion rhythm of bile acids, leading to an increase in the content of secondary bile acids, which can continuously stimulate intestinal cells. For example, deoxycholic acid and lithocholic acid directly induce DNA damage and activate signaling pathways, including epidermal growth factor receptor-, Wnt-β-catenin-, and protein kinase C pathways, thereby promoting the occurrence and development of CRC (14) (2). Cholecystectomy causes changes in the composition and abundance of the gut microbiota in both stool and tumor tissues. The main manifestations are a decline in the diversity of the microbiota, particularly, a decrease in beneficial bacteria and an increase in pathogenic bacteria (15–19), including carcinogenic bacteria, such as *Fusobacterium nucleatum*, enterotoxigenic *Bacteroides fragilis* (ETBF), *Clostridium difficile*, and *Escherichia coli*, which promote CRC development (20–23). A recent study showed that the gut microbiota of patients who underwent cholecystectomy was significantly different from that of healthy people, but similar to that of patients with CRC, which suggests that changes in the gut microbiota in patients undergoing cholecystectomy may activate CRC occurrence and progression (24).

In this article, we described the current knowledge on changes in the gut microbiota after cholecystectomy, the interaction between cholecystectomy and CRC, and potential mechanisms, aiming to clarify the role of gut microbiota alteration on the occurrence and development of CRC after a cholecystectomy.

## Epidemiological Relationship Between Cholecystectomy and CRC

In 1978, Capron was the first to report that cholecystectomy could increase the incidence of CRC (25). Subsequently, scholars worldwide conducted a series of studies on the relationship between cholecystectomy and the incidence of CRC, and two studies published in 1981 involving patients in Finland and the United States provided evidence of a significant link between cholecystectomy and CRC (10, 11).

A retrospective study in 2005 analyzed more than 8 million people on a general medicine research database in the United Kingdom and found that cholecystectomy was associated with increased risk of colon cancer, but not rectal cancer (12). Some meta-analyses have also indicated a correlation between cholecystectomy and the increasing risk of CRC. Among these, one analysis included ten cohort studies which indicated a strong correlation between the proximal colon with a history of cholecystectomy and carcinogenesis (13, 26, 27). These results indicate that a history of cholecystectomy is closely associated with the occurrence and progression of CRC.

## Effect of Cholecystectomy on Gut Microbiota

Trillions of microorganisms, such as bacteria, viruses, fungi, and other life forms, live inside every person. Various organs show distinct microbial inhabitants, but the inhabitants that have drawn the most attention are those in the colorectum (28). The gut microbiota is a key player in physiological activities, including metabolism of food residues, synthesis of micronutrients (such as vitamins), metabolism of primary bile acids, synthesis of secondary bile acids, regulation of immune responses, and metabolism and production of butyric acid and other substances which provide substances for epithelial cell renewal and mucosal integrity maintenance (29). Of note, the gut microbiota is related to the development of a wide range of digestive diseases, such as IBD, irritable bowel syndrome, and CRC (30–32).

Cholecystectomy induces dramatic changes in intestinal microecology, including the composition and function of the gut microbiota. The changes in the gut microbiota after cholecystectomy are shown in **Table 1** (24, 33–37). At the phylum level, the abundance of Fusobacteria increased, whereas that of Proteobacteria decreased. Other phyla, including Bacteroidetes, Firmicutes, and Actinobacteria, showed distinct variations in different studies. Interestingly, the changes in bacterial abundance of Firmicutes and Actinobacteria were similar in all studies, in contrast to the alteration in the bacterial abundance of Bacteroidetes. Different *Bacteroides* species affect the health of the host in different ways (38–40). For example, ETBF can induce colitis and promote the occurrence of intestinal tumors, while other species, such as *Bacteroides vulgatus* and *Bacteroides fragilis*, are associated with the protection of the intestinal barrier. At the genus level, existing research has not reached a consensus. Genera reported with increasing abundance mainly include *Anaerostipes*, *Dorea*, *Clostridium*, *Mogibacterium*, *Flavonifractor*, *Shigella*, and *Escherichia*, whilst those with reduced abundance include *Paraprevotella*, *Prevotella*, *Barnesiella*, *Alistipes*, *Faecalibacterium*, *Haemophilus*, and *Desulfovibrio*. Few studies have focused on the species level; *Blautia obeum*, *Veillonella parvula*, *Bacteroides ovatus*, *Parabacteroides distasonis*, and *Fusobacterium varium* were found to increase, and *Eubacterium rectale*, *Roseburia faecis*, and *Bifidobacterium adolescentis* were reported to decrease.

**Table 1:** Comparison of gut microbiota between patients who have undergone cholecystectomy and healthy individuals.

	Country (Author & Year)	Sample size	Sequencing method	Changes of gut microbiota after cholecystectomy
1	Israel (Keren et al., 2015) (33)	20	16S rRNA	The diversity of microbiome is basically stable. **Phylum** *Bacteroides*↑ **Family** *Bacteroides*, *Parabacteraceae*↑
2	China (Wang et al., 2018) (34)	135	16S rRNA	The diversity of microbiome declined. **Phylum** *Actinomycetes*, *Firmicutes*↑ *Bacteroides*, *Proteobacteria*↓ **Genus** *Bifidobacterium*, *Dallella*, *Anaerobic*↑ *Palapuella*, *Prevotella*, *Barnesella*, *Alternaria*, *Desulfovibrio*↓
3	Korea (Yoon et al., 2019) (35)	108	16S rRNA	The diversity of microbiome declined. **Phylum** *Firmicutes*↑ *Bacteroides*↓ **Species** *Broutella ovale*, *Veillonella parvula*↑
4	China (Ren et al., 2020) (24)	104	16S rRNA	The diversity of microbiome increased. **Phylum** *Bacteroides*, *Fusobacteria*↑ *Firmicutes*, *Actinomycetes*↓ **Genus** *Prevotella*↑ *Faecalibacterium*↓ **Species** *Bacteroides ovatus*, *Parabacteroides diundi*, *Fusobacterium proteus↑* *Eubacterium rectale, Roseburia faecis*, *Bifidobacterium adolescentis↓*
5	Germany (Frost et al., 2021) (36)	1968	16S rRNA	The diversity of microbiome declined. **Genus** *Clostridium XIVa, Flavonoids, Clostridium* *difficile, Escherichia, Shigella*↑ *Faecalibacterium*, *Haemophilus*↓

It was widely reported that *Escherichia* and ETBF had increased in abundance of CRC patients, promoting CRC development through damaged DNA, and produced toxins. While beneficial bacteria, including *Alistipes* and *Faecalibacterium*, which can produce active metabolites, such as butyrate and folic acid and inhibit the occurrence and development of CRC, were significantly reduced in patients with cholecystectomy history (24, 33–37). Due to differences in race, diet, and experimental conditions, changes in the gut microbiota after cholecystectomy are inconsistent. However, all the relevant studies confirm that alterations in the gut microbiota promote CRC occurrence and progression.

**Figure 1:**
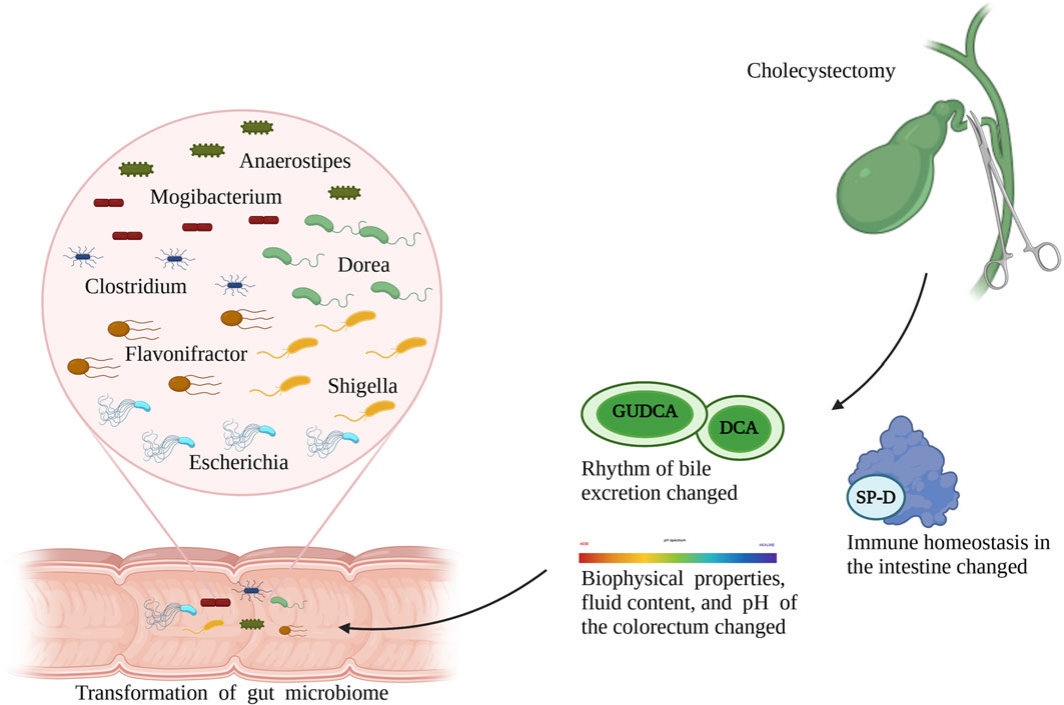
Role of cholecystectomy in the alternation of gut microbiota.

Transformation of the gut microbiota after cholecystectomy can be attributed to the following reasons (**Figure 1**): first, bile excretion regulation weakens or disappears after cholecystectomy; as a result, the bile flows into the intestine continuously (41). This changed pattern is conducive to the growth of bacteria that metabolize bile acid or live through bile-dependent fat decomposition but has adverse effects on the growth of other bacteria, thereby reshaping the gut microbiota. For example, experiments have shown that deoxycholic acid inhibits the growth of *Lactobacillus*, *Bifidobacterium*, and other bile-sensitive bacteria (42). Second, cholecystectomy alters bowel movements by changing the biophysical properties, fluid content, and pH of the colorectum, thereby providing favorable or harmful growth conditions for certain bacteria. For example, persistent secretion of bile, which is alkaline, after cholecystectomy, increases the pH value in the intestines, thereby inhibiting the proliferation of acidic-adapted bacteria, including *Lactobacillus* and *Bifidobacterium*. Third, changes in immune homeostasis in the intestines after cholecystectomy should be considered. For example, surfactant protein D, an important substance secreted by the gallbladder, can be transported to the intestinal lumen with the entered bile and inhibits the growth of symbiotic bacteria through direct binding. Cholecystectomy unavoidably decreases the level of surfactant protein D in the human intestines, which leads to disorders of bacterial and host-bacterial interactions and affects the natural environment of the gut microbiota (43).

## Role of Gut Microbiota in CRC

CRC development and progression is a multi-factor interaction, in which the role of the gut microbiota is now attracting increasing attention. A study that analyzed the Health Care Claims Database from the United States confirmed the relationship between the recurrence of CRC and disorders of the gut microbiota (44). Widespread use of antibiotics, alterations in diet, obesity, stress, and other risk factors are attributed to disorders of the gut microbiota in young people, which may partly explain their increased risk of CRC (45). A gradual increase in some bacteria and a constant decrease in some bacteria in normal, para-adenoma, adenoma, pericarcinomatous, and cancerous tissues have been found, suggesting that bacterial distribution may act as an essential factor in CRC development (46). Yu et al. conducted metagenomic sequencing of the stool samples of patients with CRC and healthy individuals and found significant differences in the composition of the gut microbiota between the two groups (47). Fecal bacterial transplantation studies were conducted, and the incidence of CRC in mice inoculated with stool samples from patients with CRC increased. The gut microbiota in the stool of patients with CRC can activate the intestinal mucosal immunity of mice and induce inflammation, so as to promote the proliferation of epithelial cells and induce the development of CRC (48). A number of clinical and animal studies have clarified the relationship between the gut microbiota and CRC and identified specific bacteria as key factors that affect the occurrence and development of CRC (19, 46, 47, 49–58), as summarized in **Table 2**, including relevant studies in the past decade, reflecting the differences in the gut microbiota between patients with CRC and healthy individuals. The diversity of the gut microbiota in patients with CRC was lower than in healthy individuals, with a decrease in beneficial bacteria and an increase in pathogenic bacteria. For example, *Fusobacterium nucleatum*, *Campylobacter*, ETBF, and *Escherichia coli* that express the polyketide synthase gene (*pks^+^ Escherichia coli*) were enriched in the intestines of patients with CRC, induced inflammation, damaged DNA, and produced toxins, thereby promoting CRC development. Beneficial bacteria, including *Bifidobacterium*, *Lachnospira*, *Alistipes*, and *Faecalibacterium*, which can produce active metabolites, such as butyrate and folic acid and inhibit the occurrence and development of CRC, were significantly reduced.

**Table 2:** Comparison of gut microbiota between patients with colorectal cancer and healthy individuals.

	Country (Author & Year)	Sample size	Sample type	Sequencing method	Changes of gut microbiota after cholecystectomy
1	China (Wang et al., 2012) (19)	102	Stool	16S rRNA	The diversity of microbiome is basically stable. **Phylum** *Firmicutes*, *Proteobacteria*, *Actinomycetes*↑ *Bacteroides*, *Fusobacteria*↓ **Genus** *Porphyromonas*, *Escherichia*, *Shigella*, *Enterococcus*, *Streptococcus*, *Peptostreptococcus*↑ *Bacteroides*, *Rossella*, *Alternaria*, *Eubacteria*, *Trichospirillum*↓
2	China (Wu et al., 2013) (50)	39	Stool	16S rRNA	The diversity of microbiome is basically stable. **Phylum** *Fusobacterium*↑ **Family** *Eubacteriaceae*, *Clostridiaceae*, *Staphylococcus*, *Enterococcus*, *Fusobacteria*, *Campylobacter*, *Porphyridaceae*↑ **Genus** *Bacteroides*, *Alternaria*, *Blautella*, *Dallella*, *Fusobacterium*, *Campylobacter*, *Escherichia*, *Shigella*, *Odorbacterium*, *Oscillatoria*, *Testa Lactobacillus*, *Rumenococcus*↑ *Rossella, Faecalibacterium*↓
3	America (Ahn et al., 2013) (49)	151	Stool	16S rRNA	The diversity of microbiome declined. **Phylum** *Bacteroides*↑ *Firmicutes*↓ **Genus** *Porphyromonas*, *Fusobacterium*, *Mirabilis*↑ *Faecococcus*, *Trichospirillum*↓
4	America (Zackular et al., 2014) (51)	60	Stool	16S rRNA	**Family** *Porphyridaceae*, *Enterobacteriaceae*↑ *Lacetospiraceae*↓ **Genus** *Porphyromonas*, *Fusobacterium*↑ *Bacteroides*↓
5	France (Zeller et al., 2014) (52)	114	Stool	Metagenomic sequencing	The diversity of microbiome is basically stable. **Phylum** *Bacteroides*, *Fusobacteria*, *Proteobacteria*↑ *Actinomycetes*, *Firmicutes*↓ **Species** *Saccharolytic Porphyromonas*, *Oral Peptostreptococcus*, *Fusobacterium nucleatum*↑
6	Australia (Feng et al., 2015) (53)	109	Stool	Metagenomic sequencing	The diversity of microbiome declined. **Genus** *Bacteroides*, *Alternaria*, *Bileophilus*, *Trichospira*, *Escherichia*, *Micromonas*, *Fusobacterium*↑ *Bifidobacterium*, *Streptococcus*, *Rumenococcus*↓
7	China (Nakatsu et al., 2015) (46)	113	Tissue	16S rRNA	**Genus** *Fusobacterium*, *Gemini*, *Peptostreptococcus*, *Micromonas*, *Streptococcus granulosus*↑ **Species** *Bacteroides fragilis*↑
8	America (Baxter et al., 2016) (54)	292	Stool	16S rRNA	**Genus** *Porphyria*, *Peptostreptococcus*, *Fusobacterium*, *Micromonas*, *Prevotella*, *Gemini*↑ **Species** *Saccharolytic Porphyromonas*, *Fusobacterium nucleatum*, *Micromonas parvum*, *Oral Peptostreptococcus*↑
9	Ireland (Flemer et al., 2017) (55)	115	Stool/ Tissue	16S rRNA	**Genus** *Bacteroides*, *Rossella*, *Rumenococcus*, *Oscillatoria*, *Porphyromonas*, *Peptostreptococcus*, *Micromonas*, *Fusobacterium*↑
10	China (Yu et al., 2017) (47)	128	Stool	Metagenomic sequencing	The diversity of microbiome declined. **Phylum** *Fusobacteria*, *Basidiomycota*↑ **Species** *Micromonas parvum*, *Oral Peptostreptococcus*, *Fusobacterium nucleatum*, *Bacteroides fragilis*, *Solobacterium moorei*↑
11	Saudi Arabia (Alomair et al., 2018) (56)	58	Tissue	Metagenomic sequencing	The diversity of microbiome is basically stable. **Genus** *Peptostreptococcus, Porphyromonas, Listeria, Atopobium, Burkholderia*, *Collins*, *Comamonas*, *Fusobacterium*↑
12	Japan (Yachida et al., 2019) (58)	616	Stool	Metagenomic sequencing	The diversity of microbiome increased. **Phylum** *Firmicutes*, *Fusobacteria*, *Bacteroides*↑ **Genus** *Bacteroides*, *Peptostreptococcus*↑ *Prevotella*, *Bifidobacterium*↓ **Species** *Fusobacterium nucleatum*, *Micromonas parvum*, *Streptococcus oralis*, *Solobacterium moorei*↑ *Phascolarctobacterium succinatutens*, *Desulfovibrio longreachensis*, *Atopobium parvulum*↓
13	Italy (Thomas et al., 2019) (57)	826	Stool	Metagenomic sequencing	The diversity of microbiome increased. **Genus** *Peptostreptococcus*, *Clostridium*, *Porphyromonas*, *Escherichia coli*↑ *Bifidobacterium*, *Trichospirillum*, *Alternative Mycobacterium*↓ **Species** *Fusobacterium nucleatum*, *Porphyromonas saccharolyticus*, *Micromonas parvum*, *Oral Peptostreptococcus*, *Escherichia coli*↑

**Figure 2:**
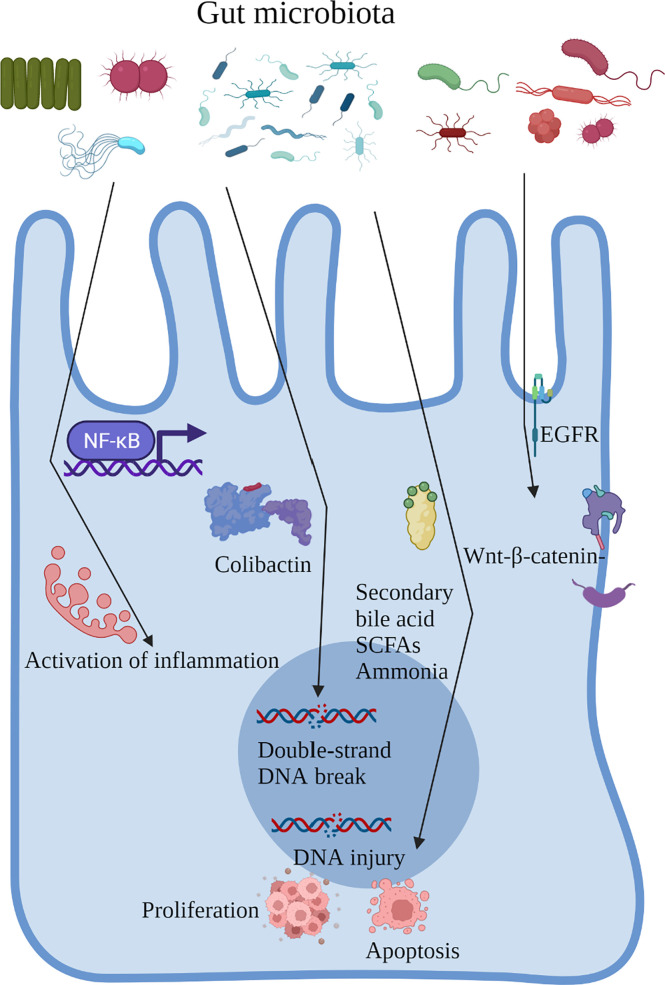
Role of the gut microbiota in the process of CRC and the carcinogenic mechanism of gut microbiota alterations after cholecystectomy.

Patients who underwent cholecystectomy and those with CRC had similar gut microbial changes, an increased abundance of pathogenic bacteria, including *Escherichia*, *Clostridium*, and *Dorea*, and a decrease in beneficial bacteria, including *Prevotella*, *Alistipes*, and *Faecalibacterium* (24, 33–37). The gut microbiota can regulate the biological behavior of the host through direct cell interactions and in a metabolite-dependent manner. In addition, intestinal inflammation caused by the gut microbiota, secretion of flora-derived factors, such as genotoxins to induce DNA damage, production of metabolites, and direct activation of carcinogenic signaling pathways are major factors in CRC development. Next, we proposed to focus on the role of the gut microbiota in the process of CRC and the carcinogenic mechanism of gut microbiota alterations after cholecystectomy (**Figure 2**).

### Inflammation and Immune Regulation

Inflammation is an established risk factor of CRC carcinogenesis. Patients with IBD are more susceptible to CRC than the general population (59, 60). Inflammation plays a key role in the development of colitis-associated cancer, even in CRC unrelated to IBD, and the levels of pro-inflammatory cytokines are increased (61). The gut microbiota has the potential to form an inflammatory microenvironment and, vice versa, inflammation may affect gut microbiota composition. Colon polyposis in Apc^min/+^ mice is accompanied by the accumulation of microorganisms in the polyps, triggering a local inflammatory response (Dennis etal. (62). Besides, defective expression of alarmin/IL-33 renders mice highly susceptible to probiotic microbiota-promoted IL-1α-dependent colitis and colitis-associated cancer (63). Gavage with stool samples from patients with CRC caused enhanced inflammation and intestinal adenoma development in a sterile mouse model (48), indicating that specific components of the gut microbiota promote the occurrence and development of CRC through the activation of inflammation. For example, enriched *Fusobacterium nucleatum* and *Escherichia coli* in the intestines of patients with CRC can activate the nuclear factor kappa B (NF-κB) signaling pathway and drive the infiltration of myeloid cells in the tumor, producing a pro-inflammatory environment that is conducive to the progression of colorectal tumors in Apc^min/+^ mice (64, 65). ETBF can trigger an inflammatory cascade involving interleukin 17, signal transducer and activator of transcription 3, and NF-κB conduction in colonic epithelial cells *via* the production of a metalloproteinase toxin, promoting the local inflammatory environment in the intestines and inducing carcinogenesis (66). Cholecystectomy increases the abundance of *Escherichia coli* and decreases the abundance of *Faecalibacterium*, which can secrete small-molecule anti-inflammatory substances to inhibit intestinal inflammation (36, 50).

Intestinal homeostasis is achieved by the continuous interaction between the intestinal microbiome and the host immune system. Once this balance is disrupted, a variety of diseases, such as IBD, appear due to immune system dysfunction (67). In mice, BFT^+^ *B. fragilis* colonization was able to induce Th-17-mediated colitis and distal CRC in an IL17-mediated NF-κB upregulation-dependent manner in the APC^min/+^ mouse model (40), as demonstrated by Chung et al. (68) who observed repressed BFT-induced tumor formation in APC^min IL17/IL17^ mice. Furthermore, it is reported that an accumulation of regulatory T-expressing cells (Treg) cells in APC^min/+^ mice after BFT colonization, which could be a trigger for IL17-mediated pro-oncogenic inflammatory responses. Certain probiotics, such as *Bifidobacterium infantis* (69) and *Bifidobacterium breve* (70), are able to activate intestinal dendritic cells (DCs) by interacting with Toll-like receptors (TLRs) and inducing retinoid metabolism, leading to the release of Foxp3^+^ Treg and type 1 regulatory T cells (Tr1) and IL-10 (71).

### Production of Genotoxins

Another carcinogenic mechanism of the gut microbiota is the production of genotoxins, which may interact with intracellular signal cascades or result in mutations by binding to particular cell surface receptors and it could also damage DNA. Colibactin is a characteristic toxin produced by *Escherichia coli*, which induces double-strand DNA breaks in intestinal cells, causing cancer through its deoxyribonuclease activity (72–74). In addition, the enriched ETBF in the intestine of patients with CRC can produce a metalloproteinase toxin, which initiates cell proliferation, activates c-Myc expression, increases polyamine metabolism, and induces DNA damage, thereby promoting the occurrence and development of CRC (75). Furthermore, *Salmonella typhi* secretes virulence protein A, which enhances the development and proliferation of colon tumors (76). Interestingly, although many genotoxins can cause tumors, recent research has indicated their potential use in cancer therapy (77). For example, *Clostridium perfringens* enterotoxin is a pore-forming toxin with selective cytotoxicity, which rapidly and effectively kills tumor cells (78). Several genotoxins have been studied as therapeutic tools for cancer, including CRC (79); however, their role as a cancer promoter is beyond doubt.

### Metabolism of Dietary Ingredients

Metabolism is an essential process in the interaction between the host and the microbiome. Genes encoded by the microorganisms can metabolize several dietary nutrients, including host-indigestible carbohydrates, such as dietary fiber, and host endogenous compounds, such as bile acids. Bacteria in the intestines produce a series of metabolites, including secondary bile acids, sulfides, ammonia, nitrosamines, and short-chain fatty acids (SCFAs), which are involved in the occurrence and development of CRC.

A substantial accumulation of primary bile acids in the intestines was discovered after cholecystectomy, and the enriched *Bacteroides ovatus* and *Parabacteroides diundi* due to cholecystectomy metabolized primary bile acids into secondary ones, which participated in cell proliferation, apoptosis, DNA injury, and other processes, promoting CRC carcinogenesis (24, 80, 81). Dietary fiber is metabolized and decomposed into SCFAs, including acetate, propionate, and butyrate, in the colon. Among them, butyrate (the most widely studied SCFA) regulates cell proliferation, apoptosis, and differentiation to inhibit CRC (82, 83). Cholecystectomy drastically reduced the abundance of intestinal bacteria responsible for metabolizing butyrate, including *Faecalibacterium* and *Roseburia faecis*, thereby decreasing the expression level of butyrate and promoting the occurrence of CRC (24). *Broutella ovale* and *Veillonella parvula*, which can activate azo reductase and produce toxic ammonia substances which promote the occurrence of CRC, were observed in patients who underwent cholecystectomy (35). Additionally, the gut microbiota can also destroy the mucus barrier function by producing sulfide, thereby intensifying the stimulation of intestinal cells (84). For instance, cholecystectomy significantly decreased the abundance of *Desulfovibrio*, producing more sulfides and leading to metabolic disorders, thereby stimulating intestinal epithelial cells and promoting carcinogenesis (34).

### Activation of Signaling Pathways

Multiple signaling pathways, such as the ﻿epithelial growth factor receptor (EGFR), Wnt/β-catenin, NF-κB, and transforming growth factor-beta pathways, are involved in CRC development. Notably, the gut microbiota activates host carcinogenic signaling pathways.

The EGFR signaling pathway is closely related to the proliferation, apoptosis, and survival of colonic epithelial cells. Activation of the EGFR signaling pathway by secondary bile acids is achieved mainly by disturbing the structure of the cell membrane (reduced membrane fluidity, altered membrane cholesterol distribution), binding to natural ligands (e.g., epidermal growth factor), or inducing calcium signaling-mediated non-dependent activation of ligands (85). Activation of EGFR activates downstream MAPK/RAS/RAF/MEX/extracellular signal-regulated kinase/proto-oncogene activator protein-1, which in turn mediates cell proliferation and activates RAS/RAF1/extracellular signal-regulated kinase signaling pathway leading to upregulation of mucin 2, also activates the phosphatidylinositol 3 kinase/Akt signaling pathway, which regulates downstream target molecules such as Caspase-8, leading to apoptosis (86, 87). When it comes to practical clinical applications, using biomarkers to target anti-EGFR treatments for metastatic CRC is well established, while the anti-EGFR antibody cetuximab is only effective against a subgroup of CRC (88, 89).

Wnt/β-linked protein signaling plays a key role not only in maintaining intestinal homeostasis but also in regulating the proliferation of CRC cells. The Wnt/β-linked protein classical signaling pathway regulates the expression of Wnt/β-linked proteins through the binding of Wnt ligands to Frizzleds receptors. The Wnt/β-linked protein can transfer to the nucleus and interact with T-cell factor and lymphatic enhancer transcription factors to modulate the transcription of downstream gene targets (survivin, Cyclin D1, and c-Myc) and affect the cell cycle pathway (90). A marked accumulation of *Fusobacterium nucleatum*, which expresses FadA adhesin on its surface, was found in patients with CRC. FadA adhesin stimulates CRC cell growth by increasing the expression of inflammatory genes, oncogenes, and transcription factors by binding to E-cadherin, activating the Wnt/β-catenin signaling pathway, and promoting the transcription of oncogenes (91). *Fusobacterium nucleatum* can also directly activate toll-like receptor signaling to promote tumor development (92).

NF-κB is a key regulator associated with inflammation and cancer on multiple levels (93). The NF-κB signaling pathway regulates many genes involved in different cellular processes, such as cell differentiation, proliferation, genomic stability, and immune responses (94), and its activation is involved in the occurrence and development of CRC. *Escherichia coli*, *Fusobacterium nucleatum*, and ETBF, which are enriched in patients with CRC, are involved in the modulation of this pathway (95). Mechanistically, *Escherichia coli* activates NF-κB through increased phosphorylation of transcription factor 65 and inhibitor of NF-κB kinase alpha, inactivation of inhibitor of NF-κB alpha, and induction of the Wnt/β-catenin pathway by upregulation of β-catenin and its downstream genes (96). The hyperactivation of NF-κB was also found in CRC tissues with abundant *Fusobacterium nucleatum*. Furthermore, ETBF activates the NF-κB pathway by stimulating intracellular interleukin 17 secretion in Apc ^min/+^ mice (68).

However, most of the existing research on cholecystectomy is focused on clinical studies, and there is a lack of in-depth mechanistic investigation after cholecystectomy. More studies are needed in the future to elucidate the changes in signaling pathways after cholecystectomy.

## Conclusion and Perspective

Epidemiological studies on CRC and cholecystectomy have proved the correlation between these two parameters and indicated that changes in the gut microbiota may be a vital intermediate link. In this review, we summarized the changes in the gut microbiota after cholecystectomy. With the variability of sequencing technologies and the complexity of bacterial populations, the conclusions were not unanimous among the studies. Despite this, the differences in the gut microbiota between patients who underwent cholecystectomy and healthy individuals have been proven, and alterations of the gut microbiota affect the development of CRC. Based on previous studies, alterations in the gut microbiota after cholecystectomy may lead to intestinal inflammation, increased metabolism of harmful substances (such as secondary bile acids), and reduction of secretion of beneficial substances (such as butyrate), resulting in the progression of CRC.

There are some potential problems with the current research on the relationship between altered gut microbiota and CRC after cholecystectomy, the results of studies on gut microbiota may vary by race, and different sequencing methods could also affect the results. In addition, alterations of gut microbiota in stool samples cannot fully reflect the tumor microenvironment. And few studies have focused on the role of beneficial bacteria in CRC. In the future, it is necessary to pay attention to the gut microbiota in cancerous and pericarcinomatous tissues and detect the changes in the local microbiome on the occurrence and development of CRC. And a decrease in abundance of beneficial bacteria should also be watched, which may be a potential therapeutic target in CRC. With the rapid development of high-throughput sequencing technology, in-depth information can be provided to understand the links between CRC, cholecystectomy, and the gut microbiota. Notably, different bacterial species of the same genus had mixed efficacy. Therefore, further studies should focus on the changes and function of the gut microbiota in patients who have undergone cholecystectomy at the species level. Hopefully, this approach will elucidate how CRC, cholecystectomy, and gut microbiota interact, allowing therapies to be targeted to individual microbiological, cancer, and lifestyle factors.

Studies on gut microbiota and CRC aim to clarify the mechanisms employed by gut microbiota in the development of CRC and further apply them to the screening, diagnosis, treatment, and prevention of CRC. For instance, Yu et al. discovered a new fecal bacterial marker (‘m3’ from a *Lachnoclostridium*) that can be used for the diagnosis of colorectal adenoma and CRC. This is superior to other stool-based tests such as fecal microbiota transplantation (FMT) and may be used for the early screening of CRC in the future (97). In addition, there are more clinical studies on techniques such as oral probiotics and FMT. Recent studies have found that the probiotic bacterium *Lactobacillus reuteri* and its produced antimicrobial compound, reuterin, can inhibit the development of CRC by depleting glutathione and inducing oxidative stress in CRC cells, resulting in protein oxidation and impaired ribosome activity. When CRC mice were orally administered *Lactobacillus reuteri*, remission, tumor shrinkage, and prolonged survival were observed in the mice (98). Recently, experimental studies on the efficacy of FMT have focused on animal models. A recent study reported that FMT from wild to laboratory mice improved host adaptation and resistance to dextran sodium sulfate/azoxymethane-induced colorectal tumorigenesis, and thus a normal gut microbiome plays a protective role in the development of CRC (99). Furthermore, the effectiveness of immunotherapy seems to be strongly influenced by the composition of the gut microbiota. Oral administration of probiotics, such as *Bifidobacterium* (100) and *Akkermansia muciniphila* (101), or FMT (102) from treatment-responsive patients greatly enhanced PD1-based immunotherapy and eliminated tumor growth through enhancing dendritic cell and T-cell responses. At present, there are several ongoing international clinical trials to validate the effect of gut microbiota on CRC chemotherapy (NCT04021589, NCT04131803, NCT01579591).

Currently, cholecystectomy is still the preferred treatment option for gallbladder stones, gallbladder polyps, and cholecystitis. Cholecystectomy is a very routine procedure and more and more patients are undergoing cholecystectomy. However, the possible induction of colorectal after cholecystectomy is getting attention, and its specific mechanism has not been elucidated yet. To clarify the specific mechanism of CRC induced after cholecystectomy can interrupt the development of CRC in a targeted way. The change of gut microbiota after cholecystectomy is an important cause of CRC, and further research on specific species of bacteria and their mechanisms will provide important methods to prevent CRC in the future. As research progresses, dietary intervention with probiotics or prebiotics, or changes in diet could potentially be an effective way to prevent CRC in patients with cholecystectomy history in the future.

## Author Contributions

All authors listed have contributed to the article and approved its publication. YM, RQ, and YZ: designing, writing, and figure plotting. CJ, ZZ, and WF: designing, funding acquisition, and review and editing. All authors contributed to the article and approved the submitted version.

## Funding

This research was supported in part by the National Key Research and Development Program of China (2018YFA0800700 and 2018YFC1003200), the National Natural Science Foundation of the P. R. of China (No. 91857115, 31925021, 81921001, 81972702 and 91959110), National multidisciplinary cooperative diagnosis and treatment capacity building project for major diseases: comprehensive diagnosis and treatment of gastrointestinal tumors, National Health and Family Planning Commission Foundation of China (Grant No. 2020YB57), and “Clinical Medicine + X” Foundation of Peking University (Grant No. PKU2021LCXQ001).

## Conflict of Interest

The authors declare that the research was conducted in the absence of any commercial or financial relationships that could be construed as a potential conflict of interest.

## Publisher’s Note

All claims expressed in this article are solely those of the authors and do not necessarily represent those of their affiliated organizations, or those of the publisher, the editors and the reviewers. Any product that may be evaluated in this article, or claim that may be made by its manufacturer, is not guaranteed or endorsed by the publisher.

## Abbreviations

CRC, colorectal cancer; ETBF, enterotoxigenic *Bacteroides fragilis*; IBD, inflammatory bowel disease; NF-κB, nuclear factor kappa B; SCFAs, short-chain fatty acids.
